# Une masse abdominale d’étiologie inattendue

**DOI:** 10.48327/mtsi.V2I1.2022.225

**Published:** 2022-03-03

**Authors:** Josaphat IBA BA, Timothée ELLA ONDO, Stéphanie NTSAME NGOUA, Ingrid NSENG NSENG, Jean Bruno BOGUIKOUMA

**Affiliations:** 1 Service de médecine interne, CHU de Libreville, BP 2228, Libreville, Gabon; 2 Service de radiologie, Hôpital d’instruction des armées, BP 2154, Libreville, Gabon

**Keywords:** Abcès, Spondylodiscite, Muscle psoas, Hôpital, Gabon, Afrique subsaharienne, Abscess, Spondylodiscitis, Psoas muscle, Hospital, Gabon, Sub-Saharan Africa

## Abstract

L’existence concomitante d’une spondylodiscite et d’un abcès du psoas chez un patient présentant des antécédents de plasmocytome doit faire l’objet d’une large recherche étiologique.

## Abstract (en)

Concomitant existence of spondylodiscitis and a psoas abscess in a patient with a history of plasmacytoma should be the subject of extensive etiological research.

## Observation

**Figure 1:**
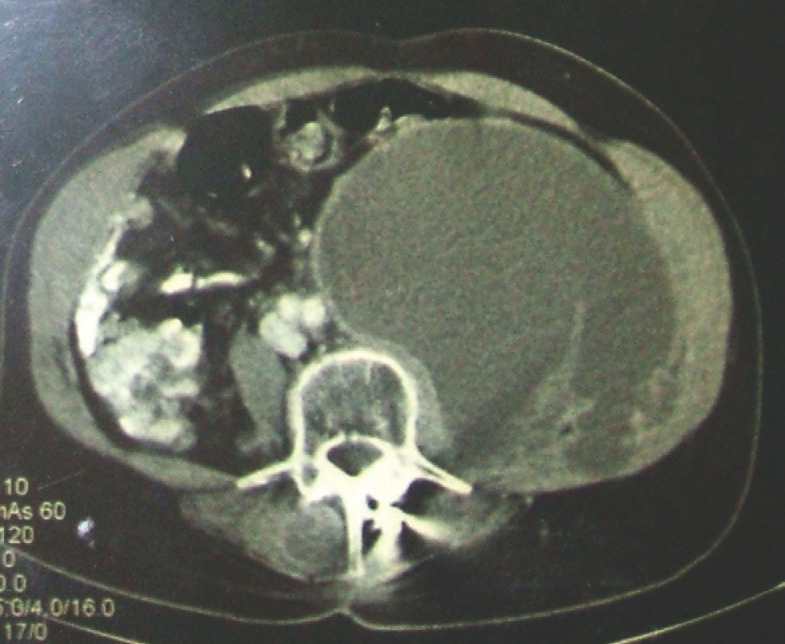
Volumineux abcès initial du psoas gauche vu en tomodensitométrie Voluminous initial left psoas abscess seen on CT scan

Un patient de 60 ans, enseignant dans le secondaire et sans souvenir d’avoir séjourné hors du Gabon depuis 3 ans, signalant des antécédents de plasmocytome solitaire vertébral de L5 traité par laminectomie + ostéosynthèse et radiothérapie au cobalt 60 (46 graves en 23 séances de L2 à S), en rémission depuis 10 ans au moment de la consultation de médecine interne, venait pour altération progressive de l’état général évoluant en l’absence de contexte fébrile, avec lombalgie basse alternant entre type inflammatoire et mécanique et raideur rachidienne. Il n’existait pas de signes cutanés, digestifs, urinaires ou pelviens. Les données pertinentes de l’examen clinique se résumaient à une raideur rachidienne avec douleur à la pression des épineuses de L4 et L5 sans déficit sensitivo-moteur ni trouble des réflexes, une masse de l’hypochondre gauche mesurant 10 centimètres de grand axe, non douloureuse et non soufflante se dissociant de la rate, des râles crépitants des deux bases et une auscultation cardiaque normale. Sur le plan biologique existait à l’hémogramme une anémie normochrome normocytaire à 10,2 g/dl, sans leucopénie ni thrombopénie, une vitesse de sédimentation à 94 mm, un bilan rénal et hépatique sans particularités. Les hémocultures étaient stériles, la sérologie VIH 1 et 2 négative. Sur le plan morphologique: la radiographie du thorax était normale; l’échographie abdominale retrouvait une masse cloisonnée du flanc gauche, mesurant 140 x 110 mm de diamètre confirmée par la tomodensitométrie abdomino-pelvienne (Fig. 1) avec rehaussement périphérique après injection de produit de contraste. La tomodensitométrie vertébrale retrouvait des images de destruction des plateaux vertébraux adjacents à l’espace L4-L5 et L5-S1, avec pincement discal et épaississement des parties molles en regard. Du fait du matériel d’ostéosynthèse, la réalisation d’une imagerie par résonance magnétique était récusée. L’échocardiographie transthoracique était sans particularités.

L’hypothèse diagnostique la plus probable est celle d’une spondylodiscite avec fistulisation dans le psoas gauche d’étiologie à préciser.

## Hypothèses Diagnostiques

- Une spondylodiscite à pyogène avec fistulisation abdominale n’est pas retenue devant la symptomatologie non bruyante avec absence de température, de porte d’entrée, d’hyperleucocytoseàl’hémogramme, degerme documenté aux différentes hémocultures. Les germes les plus fréquemment incriminés dans ce cas sont: staphylocoque doré, *Streptococcus sp., Bacteroides fragilis,* et *Escherichia coli* [3].
- Un kyste hydatique avec localisation vertébrale et du psoas doit être évoqué devant toute masse liquidienne lombaire ou iliaque. L’échographie est habituellement en faveur d’un kyste multicloisonné avec image kystique uniloculaire sans paroi propre. Il s’agit d’une parasitose dont la prévalence est élevée dans les zones d’élevage, et dont les zones d’endémie sont les pays du Maghreb. Par ailleurs, la localisation vertébrale du kyste hydatique est rare [2].
- Une endocardite avec localisations métastatiques septiques vertébrale et abdominale est peu probable devant l’absence de signes généraux, de souffle, de contexte septique, et de mise en évidence de germes aux hémocultures, et par une échocardiographie sans particularités.
- Une récidive de plasmocytome solitaire vertébral de L5, avec transformation en myélome, surinfecté avec métastases septiques nous semble peu probable car dans la revue de la littérature la progression vers le myélome multiple se produit habituellement dans les deux ans suivant le diagnostic initial.

## Diagnostic Final

**Figure 2:**
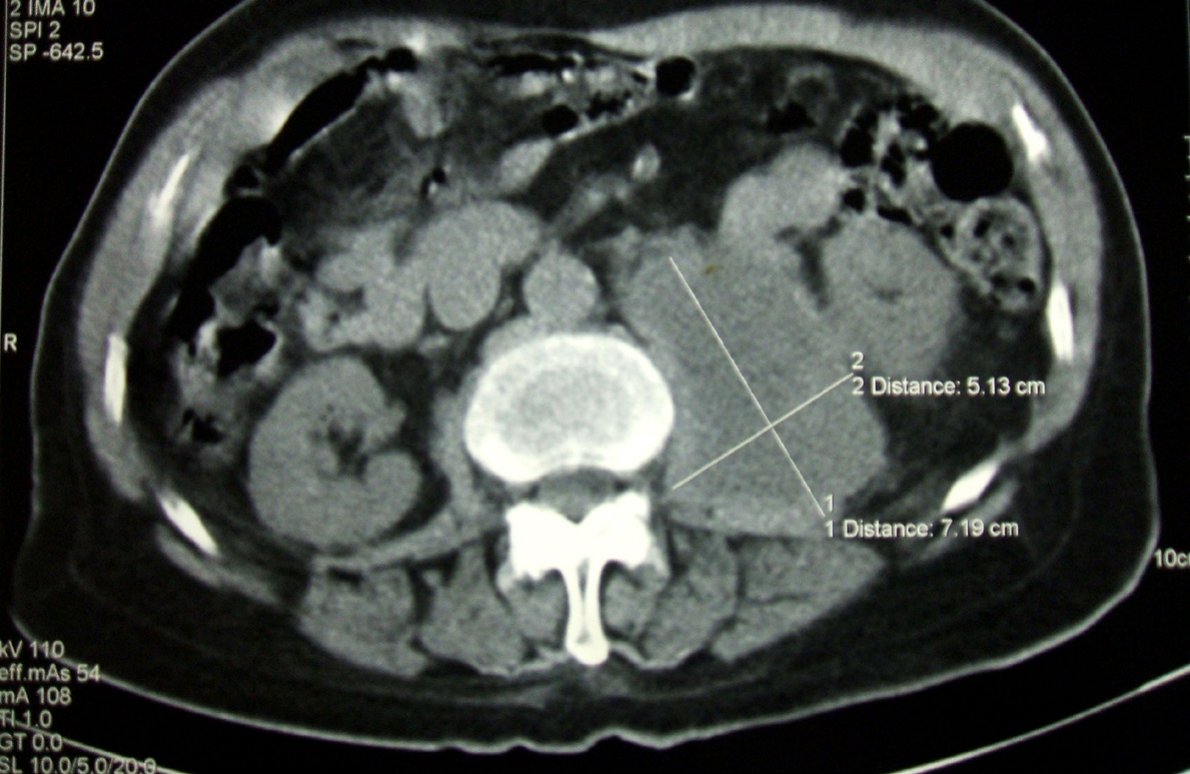
Contrôle tomodensitométrique après ponction scanner guidée CT scan control following guided puncture

**Figure 3:**
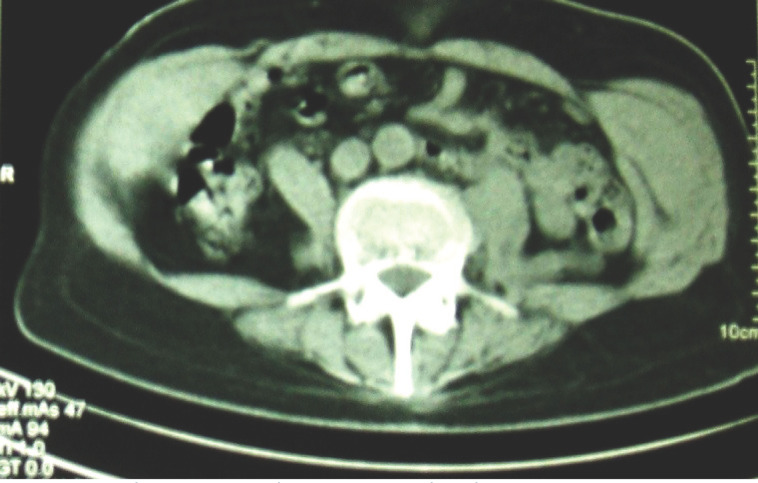
Contrôle après 14 mois de traitement antituberculeux Check-up after 14 months’ TB treatment

Il s’agissait d’une tuberculose multifocale évoquée devant: les données épidémiologiques (la tuberculose sévit à l’état d’endémie au Gabon), l’existence d’une altération progressive de l’état général malgré l’absence de signes d’imprégnation tuberculeuse notamment de fièvre, l’âge avancé du patient, l’absence d’hyperleucocytose à l’hémogramme concomitant d’un syndrome inflammatoire, l’existence d’une spondylodiscite étagée à la tomodensitométrie, et d’un abcès du psoas. L’intradermo-réaction à la tuberculine réalisée était positive à 20 mm et phlycténulaire, la ponction scanner guidée de la masse abdominale [1] ramenait 2,9 litres du caséum liquide (Fig. 2). La recherche de BK sur le liquide de ponction a été négative et il n’y a pas eu d’examen anatomopathologique. La mise sous traitement antituberculeux pendant une durée de 14 mois s’est accompagnée d’un assèchement définitif de l’abcès sans récidive (Fig. 3) et d’une consolidation des lésions de spondylodiscite.

## Liens d’intérêts

Les auteurs ne déclarent aucun lien d’intérêt.

## Contribution des auteurs

Tous les auteurs ont lu et approuvé le manuscrit final. Josaphat IBA BA et Thimothée ELLA ONDO: conception, rédaction, relecture. Stéphanie NTSAME NGOUA et Ingrid NSENG NSENG: rédaction et relecture. Jean Bruno BOGUIKOUMA: relecture.
